# Microarray Data Reveal Relationship between Jag1 and Ddr1 in Mouse Liver

**DOI:** 10.1371/journal.pone.0084383

**Published:** 2013-12-31

**Authors:** Lara A. Underkoffler, Erikka Carr, Anthony Nelson, Matthew J. Ryan, Reiner Schultz, Kathleen M. Loomes

**Affiliations:** 1 Division of Gastroenterology, Hepatology and Nutrition, The Children’s Hospital of Philadelphia, Philadelphia, Pennsylvania, United States of America; 2 Department of Pediatrics, Raymond and Ruth Perelman School of Medicine at the University of Pennsylvania, Philadelphia, Pennsylvania, United States of America; 3 Department of Medical Genetics, Kings College, London, United Kingdom; Medical University Innsbruck, Austria

## Abstract

Alagille syndrome is an autosomal dominant disorder involving bile duct paucity and cholestasis in addition to cardiac, skeletal, ophthalmologic, renal and vascular manifestations. Mutations in *JAG1*, encoding a ligand in the Notch signaling pathway, are found in 95% of patients meeting clinical criteria for Alagille syndrome. In order to define the role of *Jag1* in the bile duct developmental abnormalities seen in ALGS, we previously created a *Jag1* conditional knockout mouse model. Mice heterozygous for the *Jag1* conditional and null alleles demonstrate abnormalities in postnatal bile duct growth and remodeling, with portal expansion and increased numbers of malformed bile ducts. In this study we report the results of microarray analysis and identify genes and pathways differentially expressed in the *Jag1* conditional/null livers as compared with littermate controls. In the initial microarray analysis, we found that many of the genes up-regulated in the *Jag1* conditional/null mutant livers were related to extracellular matrix (ECM) interactions, cell adhesion and cell migration. One of the most highly up-regulated genes was *Ddr1*, encoding a receptor tyrosine kinase (RTK) belonging to a large RTK family. We have found extensive co-localization of Jag1 and Ddr1 in bile ducts and blood vessels in postnatal liver. In addition, co-immunoprecipitation data provide evidence for a novel protein interaction between Jag1 and Ddr1. Further studies will be required to define the nature of this interaction and its functional consequences, which may have significant implications for bile duct remodeling and repair of liver injury.

## Introduction

Alagille syndrome (ALGS) is an autosomal dominant disorder involving bile duct paucity and cholestasis in addition to cardiac, skeletal, ophthalmologic, renal and vascular manifestations. Mutations in *JAG1*, encoding a ligand in the Notch signaling pathway, are found in 95% of patients meeting clinical criteria for ALGS, and a small number of patients have mutations in *NOTCH2* [1]–[3]. In order to define the role of *Jag1* in the bile duct developmental abnormalities seen in ALGS, we previously created a *Jag1* conditional knockout mouse model [4]. Conditional ablation of *Jag1* in hepatoblasts results in normal bile duct development, but mice heterozygous for the *Jag1* conditional and null alleles demonstrate abnormalities in postnatal bile duct growth and remodeling, with portal expansion and increased numbers of malformed bile ducts. In this study we report the results of microarray analysis and identify genes and pathways differentially expressed in the *Jag1* conditional/null livers as compared with littermate controls.

In the initial microarray analysis, we found that many of the genes up-regulated in the *Jag1* conditional/null mutant livers were related to extracellular matrix (ECM) interactions, cell adhesion and cell migration. One of the most highly up-regulated genes was *Ddr1*, encoding a receptor tyrosine kinase (RTK) belonging to a large RTK family. Ddr1 is unusual in that it is activated by various collagen ligands as opposed to the classical RTK activation via soluble growth factors [5], and receptor activation by extracellular collagen might provide a mechanism for cell-to-ECM communication [6]. Information from published literature regarding Ddr1 structure and function led us to hypothesize that Jag1 and Ddr1 might interact in the extracellular matrix during normal bile duct development and remodeling. First, reported functions of Ddr1 include cell growth, migration, adhesion and branching tubulogenesis [5], [7]–[9], all properties that are known to be crucial for the normal growth and remodeling of bile ducts. In addition, loss of *Jag1* or *Ddr1* in a mouse model leads to similar inner ear phenotypes. Ddr1^−/−^ mice exhibit hearing loss by 2 months of age as a result of progressive deterioration of the sensory epithelium within the organ of Corti, as well as morphological defects in the cells that make up the stria vascularis including strial cells, basal cells, marginal cells and intermediate cells. Overall, Ddr1 function is thought to be essential for maintaining tissue architecture and controlling collagen deposition in the inner ear [8]. The Notch ligand *Jag1* also plays a role in inner ear development of the mouse [10]. The headturner (Htu) mouse, a *Jag1* loss-of-function mutant, displays a reduction in the number of outer hair cells in the organ of Corti as well as a significant increase in the number of inner hair cells [10]. Finally, a recent study has identified Notch1 as a direct target of collagen-mediated Ddr1 activation [11]. Up-regulation of Hes1 was demonstrated in breast cancer cells as well as colorectal carcinoma cells, indicating canonical Notch signaling is activated through this interaction [11].

In this study, we report the results of microarray analyses to identify differentially expressed genes and pathways in *Jag1* conditional/null livers, which reveal up-regulation of many genes related to fibrosis and ECM interactions. In addition, we present protein expression data showing extensive co-localization of Jag1 and Ddr1 in bile ducts and blood vessels in postnatal liver. Finally, co-immunoprecipitation of the proteins provides evidence for a novel protein interaction between Jag1 and Ddr1.

## Materials and Methods

### Mice and Breeding

Genetic strains used in these experiments include *Jag1^loxP^* [4], *Jag1^dDSL^* [12], and the Alfp-Cre transgenic line [13]. The *Jag1^loxP^* and Alfp-Cre mice were initially maintained on a C57Bl6/SvEv background, and now have been backcrossed to C57Bl/6J for >10 generations. The *Jag1^dDSL^* mice were maintained on a C57Bl/6J background (backcrossed >10 generations). Genotyping for all mice was performed by PCR analysis using genomic DNA isolated from the tail tip of weanling mice. All procedures involving mice were conducted in accordance with federal guidelines and approved Institutional Animal Care and Use Committee protocols. All animals received humane care according to the criteria outlined in the “Guidelines for the Care and Use of Laboratory Animals.”

### Ethics Statement

This study was carried out in strict accordance with the recommendations in the Guide for the Care and Use of Laboratory Animals of the National Institutes of Health. The protocol was approved by the Institutional Care and Use Committee at The Children’s Hospital of Philadelphia (Approval number 2010-11-431). All attempts were made to minimize suffering.

### Microarray Analysis

RNA was generated from 3 *Jag1* conditional/null mutant adult liver samples, each with a littermate control. Samples were obtained from mice at 12 weeks, 8 weeks and 4 weeks of age. Biotin-labeled cRNA probes were synthesized and hybridized to Affymetrix® Mouse Genome 430A 2.0 GeneChip Arrays. Data were analyzed with the ArrayAssist software (Stratagene) to generate GC-RMA absolute expression values, which were log-transformed and subjected to a t-test to identify differentially-expressed candidate genes. Student t-test was performed between the 2 groups of samples (n = 3 for mutants and controls) for each probe set on the array. Genes with a fold change greater than or equal to 2.5 and an uncorrected p-value less than 0.05 were selected for further study. Data were also analyzed by Spotfire and Ingenuity Pathway Analysis. The microarray dataset has been submitted to Gene Expression Omnibus with accession number GSE46577.

### PCR Array Analysis

In order to validate the results of the microarray experiment, custom PCR arrays (SA Biosciences®, Valencia, CA) were designed, including 8 genes of interest identified as being differentially expressed in the mutant liver samples by the criteria listed above (see Table 1 for a complete gene list and Table 2 for genes included on the custom array) and *Hprt* as a housekeeping gene. RNA and cDNA were prepared from *Jag1* conditional/null and control mouse livers obtained at 4, 8 and 12 weeks. Changes in gene expression were calculated using the ΔΔCT method, and results expressed as fold change relative to selected housekeeping genes. Samples from each mutant group were compared to their respective littermate controls.

**Table 1:** Genes Up-regulated in Mutant Liver Samples, with Fold Change >2.5.

Unigene	Gene Symbol	Gene Title	Fold Change
Mm.29798	Cd34^a^ *	CD34 antigen	5.9085
Mm.286834	Bicc1	bicaudal C homolog 1 (Drosophila)	5.5558
Mm.182785	Emp1*	epithelial membrane protein 1	5.5109
Mm.28935	Dpt^b^ *	dermatopontin	5.3057
\N	LOC672274*	similar to Transcription factor SOX-4	4.4639
Mm.10760	Osmr^c^	oncostatin M receptor	4.1651
Mm.7562	Col6a3^b^	procollagen, type VI, alpha 3	4.0085
Mm.260227	Pak1	p21 (CDKN1A)-activated kinase 1	3.9983
Mm.18888	Lum^b^	lumican	3.7683
Mm.2408	C3ar1*	complement component 3a receptor 1	3.4893
Mm.250492	Loxl1^b^	lysyl oxidase-like 1	3.4667
Mm.5021	Ddr1^d^	discoidin domain receptor family, member 1	3.4395
Mm.4146	Pdgfrb^c^ *	platelet derived growth factor receptor, beta	3.4275
Mm.211535	Olfml3	olfactomedin-like 3	3.4268
Mm.252321	Synpo	synaptopodin	3.4033
Mm.42163	Casp12*	caspase 12	3.3781
Mm.112933	Adamts5^e^	a disintegrin-like and metallopeptidase/thrombospondin	3.3192
Mm.4691	Nid1^b^	nidogen 1	3.3091
Mm.200916	Gpx3	glutathione peroxidase 3	3.1160
\N	LOC637870*	similar to Nedd4 WW binding protein 4	3.0118
Mm.20897	Clca1*	chloride channel calcium activated 1	2.9732
Mm.432071	Gdf10*	growth differentiation factor 10	2.9487
Mm.221403	Pdgfra^c^ *	platelet derived growth factor receptor, alpha	2.9136
Mm.13787	Cp*	ceruloplasmin	2.8745
Mm.292100	Fgl2*	fibrinogen-like protein 2	2.6800
Mm.7214	Anxa3	annexin A3	2.6550
Mm.206505	Timp2^e^	tissue inhibitor of metalloproteinase 2	2.6139
Mm.10125	Ift81*	intraflagellar transport 81 homolog	2.5475

^a^ cell adhesion,

^b^ ECM structural proteins,

^c^ Fibrosis-related receptor,

^d^ ECM interacting proteins,

^e^ ECM remodeling proteins.

genes with p-values<or = 0.01.

Microarray analysis of Jag1 conditional/null adult mouse livers as compared to controls. Genes with fold changes greater than 2.5 and uncorrected p-values of <0.05 are listed and show expression increases in genes regulating extracellular matrix interactions and remodeling as well as cell adhesion and fibrosis.

**Table 2:** Validation of Microarray Data.

Microarray Data		PCR Array Data		PCR Array Data	
4,8,12 week samples		4,8,12 week samples		4 week samples only	
Gene Symbol	Fold Change	Fold change	p-value	Fold Change	p-value
CD34	5.91	3.36	0.03*	4.61	0.04*
Ddr1	3.44	3.85	0.13	6.74	0.007**
Dpt	5.31	3.33	0.03*	3.89	0.02*
Loxl1	3.47	3.99	0.07	4.31	0.02*
Lum	3.77	3.29	0.04*	4.81	0.04*
Nid1	3.31	3.30	0.15	3.15	0.03*
Osmr	4.17	2.33	0.20	3.61	0.07
Pdgfrb	3.43	2.69	0.09	2.63	0.06

A custom PCR array was used to validate the fold changes found in the original microarray experiment. At the 4 week time point, 6 of the 8 genes investigated reached statistical significance, indicating the increased severity of the phenotype at the earlier time point. Ddr1 was the most highly up-regulated gene found with the most statistically significant p-value, thus prompting further investigation of the possible relationship between Jag1 and Ddr1. (* = p≤0.05; ** = p≤0.01).

Differential gene expression was also assessed between mutant and control livers using the Extracellular Matrix and Adhesion Molecule PCR Array (SA Biosciences®, Valencia, CA). This real time PCR-based array measures expression of 84 ECM-related genes. RNA and cDNA were prepared from *Jag1* conditional/null and control mouse livers obtained at 12–16 weeks (n = 3) and 4 weeks of age (n = 3). Changes in gene expression were calculated using the ΔΔCT method, and results expressed as fold change relative to selected housekeeping genes. Samples from each mutant group were compared to averages of controls in all age groups (n = 8).

### Sirius Red Staining

Livers were collected and fixed in buffered formalin and paraffin embedded, followed by sectioning at 5 µm-thickness. Sirius Red staining was performed as in [14].

### Immunofluorescence

Livers were collected at appropriate time points and embedded in OCT compound, snap frozen and stored at −80°C. Immunofluorescent staining was performed on 5 µm frozen sections of mouse liver. Slides were fixed in cold acetone and washed in PBS. Sections were incubated at room temperature in 0.25% triton-X for antigen retrieval and blocked with Pro-block from ScyTek laboratories (Logan, UT). Primary antibodies were applied at appropriate dilutions and slides were incubated from 2 hours to overnight. Jag1 sc-6011 from Santa Cruz Biotech was used at 1∶50 dilution, Ddr1 sc-532 also from Santa Cruz Biotech was used at 1∶100 dilution and CK19 TROMA-III antibody (Developmental Studies Hybridoma Bank, University of Iowa) was used at 1∶75 dilution. Slides were washed in PBS and incubated with secondary antibodies for 1 hour at room temperature in darkened conditions. Secondary antibodies used were Dylight® 649-conjugated donkey anti-goat at a dilution of 1∶400, Cy2-conjugated donkey anti-rat at a dilution of 1∶400 and Cy3-conjugated donkey anti-rabbit at a dilution of 1∶600 (all from Jackson Immunoresearch, West Grove, PA). Nuclei were stained with DAPI for 20 seconds. Finished specimens were viewed with the Olympus BX-51 fluorescent microscope (Center Valley, PA).

### Co-immunoprecipitation and Western Blot

Immunoprecipitation was performed using the Co-Immunoprecipitation kit from Pierce® ThermoScientific (Rockford, IL). Antibodies were first de-salted and cleaned using the Pierce antibody clean–up kit. The antibodies were then immobilized through the covalent coupling onto an amine-reactive resin. Liver lysates were made using PBS with 5 mM EDTA, 1 mM CaCl_2_, 0.02% sodium azide plus appropriate protease- and phosphatase-inhibitors. Approximately 200 µg of lysate per antibody were pre-cleared using a deactivated control resin, supplied by the manufacturer, by incubating for 1 hour at 4°C. Binding of target and prey proteins were carried out at the same temperature overnight. Bound products were eluted following several washes to remove unbound proteins. Western analysis followed to identify IP and Co-IP products.

The Jag1 sc-6011 and Ddr1 sc-532 rabbit antibodies manufactured by Santa Cruz Biotechnology, Inc (Santa Cruz, CA) were used at a 1∶200 dilution for western blots. Approximately 25 µg of each antibody was coupled to the AminoLink resin (Pierce® ThermoScientific, Rockford, IL). In addition to the proteins of interest, an irrelevant antibody (SC-324 rabbit polyclonal anti-Tie2, Santa Cruz Biotechnology, Santa Cruz, CA) was also coupled and used as a negative control in the western analysis.

### Jag1 Real-time PCR on Whole Newborn Liver

Total RNA was prepared from whole liver newborn mutant and control livers. cDNA was prepared from 4 µg of total RNA using the high capacity RNA-to-cDNA kit from Applied Biosystems (Carlsbad, CA). Assuming 100% conversion of RNA to cDNA, 100 ng of cDNA was used per 20 µl PCR reaction. Each sample was prepared in quadruplicate using TaqMan Gene Expression assays for the housekeeping gene, *Hprt1* (Mm00446968_m1) as well as for *Jag1* (Mm00496904_m1). The *Jag1* probe is a made-to-order probe that encompasses the region of the targeted *Jag1* deletion including exons 4 and 5. The plates were run using an Applied Biosystems 7300/7500 Real-Time PCR Standard System using the default program of 50 degrees for 2 minutes, 95 degrees for 10 minutes and 40 cycles of 95 degrees for 15 seconds, 60 degrees for 1 minute. Data were analyzed using ΔΔCT method with Applied Biosystems 7500 Real-Time PCR System Software v1.4.

## Results

### Gene Expression Analysis Reveals Upregulation of Fibrosis-related Genes in Mutant Livers

After our initial studies identified abnormalities in postnatal bile duct growth and remodeling in mice heterozygous for the *Jag1* conditional and null alleles, we sought to characterize differentially expressed genes and pathways in mutant and control mouse livers [4]. We hypothesized that specific genes and pathways would be up- or down-regulated in the mutant mouse livers, and that the identification of these genes would improve our understanding of the pathways involved in postnatal bile duct growth and remodeling, as well as elucidate potential Notch pathway interactions. In order to assess global changes in gene regulation across different time points, we elected to study gene expression by microarray analysis in *Jag1* conditional/null mouse livers at 4, 8 and 12 weeks of age, as compared with littermate control samples. Biotinylated probes were generated for each sample and hybridized to Affymetrix® Mouse Genome 430A 2.0 GeneChip Arrays as described in detail in Materials and Methods. The results of this analysis are presented in Table 1.

**Figure 1:**
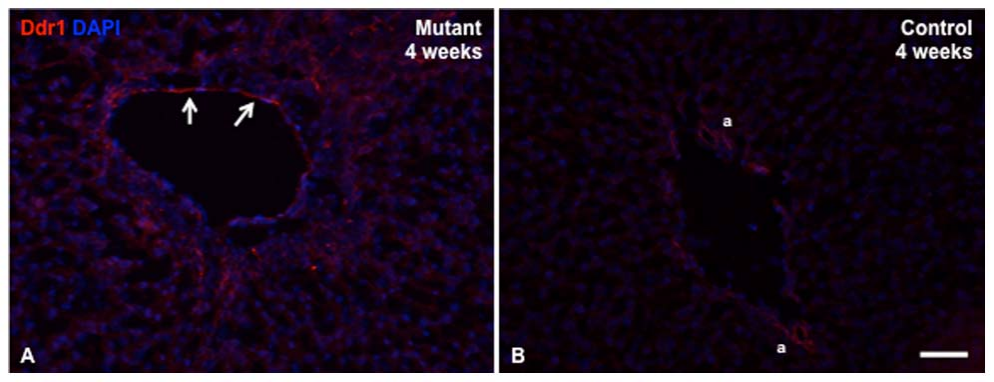
Ddr1 protein expression is increased in mutant liver at 4 weeks of age. A. Immunofluorescence staining shows increased expression of Ddr1 protein in the periportal region of the *Jag1* conditional/null mouse liver at 4 weeks of age. Arrows depict Ddr1 protein expression in the portal vein endothelium. B. In control liver, Ddr1 protein staining is visible in the arteries (a), but to a lesser extent in the peri-portal area. (Scale bar in [B] = 25 μ for [A] and [B]).

Table 1 lists genes found to have increased expression levels in the mutant liver samples compared to controls, with a fold change greater than or equal to 2.5, and an uncorrected p-value of <0.05. Interestingly, many of the genes identified in this analysis are related to fibrosis, extracellular matrix interactions and remodeling, or cell adhesion, all of which are important in the process of bile duct remodeling. For example, *CD34*, up-regulated in the mutant liver samples with a fold change of 5.9, encodes a cell surface glycoprotein that functions as a cell-cell adhesion factor [15]. Similarly, *Dpt* (fold change 5.3) encodes dermatopontin, an extracellular matrix protein that may function in cell-matrix interactions and matrix assembly. Other up-regulated genes include those encoding components of the ECM and basement membrane (*Col6a3* and *Nid1*), as well as those known to bind collagen, such as *Ddr1* and lumican. In order to validate the results of the microarray, we designed a custom PCR array including eight of the most highly up-regulated genes and *Hprt* as a housekeeping gene for normalization (Table 2). We chose eight of the top twenty upregulated genes to study further on the PCR array based on their predicted functions in cell adhesion, ECM structure or interaction, and fibrosis (Tables 1 and 2). By using the PCR array approach as opposed to individual real time PCR assays, we were able to validate our findings in a single experiment. Using the same 4, 8 and 12-week samples as those tested on the microarray, the PCR array showed upregulation in all 8 genes with similar fold changes, and reached statistical significance for 3 of the genes (*CD34, Dpt* and *Lum*; p<0.05). We also examined differential expression of these 8 genes specifically at 4 weeks of age (n = 3), and found that all of the genes were up-regulated in the mutant samples with 6 of the 8 attaining statistical significance with p-values <0.05. Of interest, *Ddr1* was the most highly expressed gene at 4 weeks of age, with a fold change of 6.74 and a p-value of 0.007. Ddr1 protein levels are also increased in the mutant livers at 4 weeks age as compared with controls (Figure 1).

**Figure 2:**
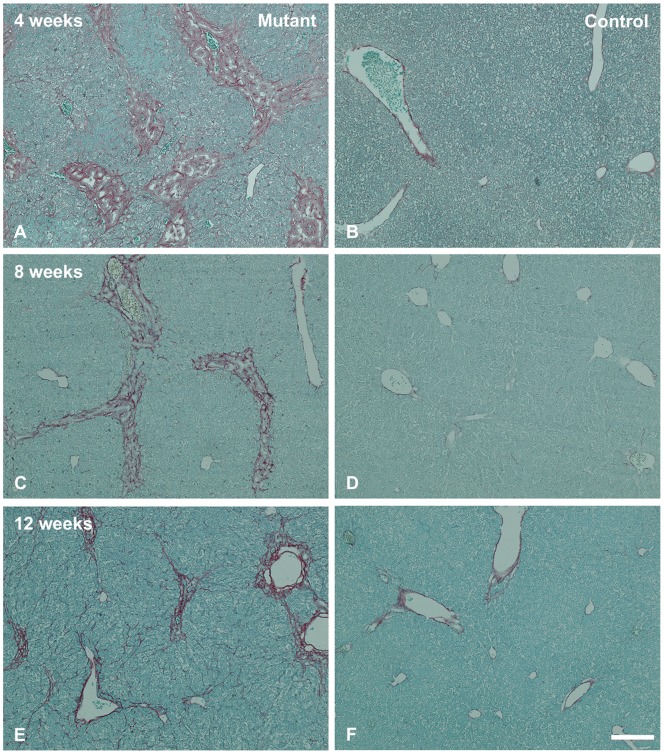
Sirius Red staining demonstrates hepatic fibrosis in *Jag1* conditional/null livers. A. Significant portal expansion with bridging fibrosis is visible in the *Jag1* conditional/null mutant liver at 4 weeks of age. B. Control liver does not show fibrosis at 4 weeks of age. C. Portal expansion is less prominent in the mutant liver at 8 weeks of age, but bridging fibrosis is still present in some area. D. Control liver shows no fibrosis at 8 weeks of age. E. At 12 weeks of age, fibrosis is improving in the *Jag1* conditional/null mutant livers. F. Control liver at 12 weeks of age shows no fibrosis. (Scale bar in [F] = 100 μ for [A] through [F]).

Given the increased expression of fibrosis- and ECM-related genes in the *Jag1* conditional/null liver samples, we assayed for the presence of fibrosis in mutant and control livers at postnatal time points between 4 and 12 weeks of age (Figure 2). At 4 weeks of age, Sirius Red staining demonstrates significant expansion of the portal tracts in the mutant liver with clear bridging fibrosis (Figure 2A) as compared with the littermate control (Figure 2B). Sirius red staining at earlier stages shows that fibrosis is detectable in the mutant livers as early as 2 weeks of age (data not shown). At 8 weeks of age, the degree of fibrosis in the mutant livers is less severe than that seen at 4 weeks of age (Figure 2C). By 12 weeks of age, the fibrosis appears to be resolving in the mutant livers, with mildly expanded portal tracts and a few thin bands of connective tissue extending between portal tracts (Figure 2E). This is in keeping with our prior finding of mild portal expansion at 12 weeks of age in the mutant livers [4]. At both 8 and 12 weeks of age, control livers show no significant fibrosis (Figure 2D and F). In order to investigate the striking early onset of hepatic fibrosis at 4 weeks of age in the *Jag1* conditional/null mutant animals, we performed PCR array analysis specifically for genes related to the extracellular matrix (ECM Array, SA Biosciences). Of the 84 ECM-related genes included on the array, 24 were significantly up-regulated in the *Jag1* conditional/null mutant livers at 4 weeks of age with p-values <0.05 (9 with p-values <0.01, Table 3). Highly up-regulated genes include *Col1a1* and *Vcan*, both encoding structural proteins within the ECM. E-selectin (fold change 18.4) is a cell adhesion molecule expressed only on cytokine-activated endothelial cells and involved in inflammation, indicating global liver injury. In addition, elevated expression of multiple matrix metallopeptidases indicates active matrix remodeling. Only one gene, *Ecm1,* encoding Extracellular Matrix Protein 1, is significantly downregulated with a fold change of −4.06. Ecm1 has been reported to inhibit the proteolytic activity of Mmp9 [16]; therefore its downregulation may be in keeping with the overall activation of matrix metallopeptidases and other matrix remodeling proteins in our model. Interestingly, by 12 to 16 weeks of age, gene expression levels had returned to baseline, suggesting that wound repair and remodeling were complete (Table 3).

**Table 3:** Timecourse of Extracellular Matrix & Adhesion PCR Array Gene Expression.

Gene Symbol	Gene Title	Fold Change		
		12–16 weeks	4 weeks	p-value*
Cd44	CD44 antigen	1.19	2.78	0.044
Cdh3	Cadherin 3	−1.38	3.69	0.007
Col1a1	Collagen, type I, alpha 1	−1.26	22.14	0.020
Col3a1	Collagen, type III, alpha 1	−2.00	7.95	0.051
Vcan	Versican	−1.18	15.12	0.003
Ecm1	Extracellular matrix protein 1	−1.38	−4.06	0.003
Fbln1	Fibulin 1	1.02	4.38	0.031
Icam1	Intercellular adhesion molecule 1	1.30	2.88	0.032
Itgam	Integrin alpha M	1.47	5.52	0.006
Itgax	Integrin alpha X	1.91	5.35	0.007
Mmp11	Matrix metallopeptidase 11	1.03	3.20	0.005
Mmp13	Matrix metallopeptidase 13	1.65	6.32	0.020
Mmp15	Matrix metallopeptidase 15	1.72	3.33	0.046
Mmp2	Matrix metallopeptidase 2	1.13	8.26	0.023
Mmp3	Matrix metallopeptidase 3	−1.75	5.39	0.052
Mmp7	Matrix metallopeptidase 7	2.34	4.61	0.023
Mmp8	Matrix metallopeptidase 8	1.83	17.98	0.000
Mmp9	Matrix metallopeptidase 9	1.74	5.56	0.018
Sele	Selectin, endothelial cell	1.16	18.42	0.007
Sell	Selectin, lymphocyte	1.57	3.31	0.053
Spp1	Secreted phosphoprotein 1 (Osteopontin)	−1.68	8.93	0.001
Thbs1	Thrombospondin 1	1.17	8.64	0.016
Thbs2	Thrombospondin 2	1.29	4.13	0.019
Thbs3	Thrombospondin 3	1.46	5.34	0.033
Tnc	Tenascin C	−1.43	10.82	0.009

p-values listed are for the 4 week data only.

Real-time PCR was employed using the Extracellular Matrix & Adhesion molecules PCR Array to investigate the changes in gene expression related to fibrosis and injury over time. The data support a repair mechanism is likely active between the peak fibrotic phenotype at 4 weeks of age and 12–16 weeks of age.

**Figure 3:**
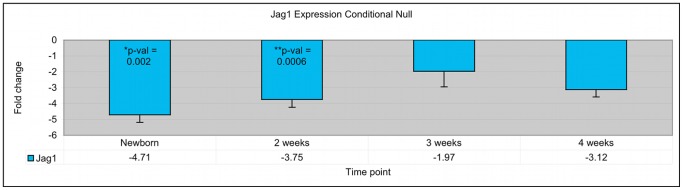
Time course of *Jag1* gene expression in *Jag1* conditional/null mouse liver samples. Measurement of *Jag1* expression in whole mouse liver by real time PCR reveals that *Jag1* expression is significantly decreased in *Jag1* conditional/null mutant livers at newborn and 2 week time points. At 3 and 4 weeks of age, *Jag1* expression is not significantly decreased in whole liver, possibly corresponding to increased *Jag1* expression in other cell types within the liver.

In order to characterize the time course of *Jag1* expression in the *Jag1* conditional/null mutant livers, we performed real time PCR analysis on whole liver samples between newborn and 4 weeks of age (Figure 3). At the newborn timepoint, *Jag1* expression was significantly downregulated in the mutant livers with a fold change of −4.71 (p = 0.002), in keeping with ablation of *Jag1* from hepatoblasts. Similarly, at 2 weeks of age, *Jag1* expression is diminished in the mutant livers with a fold change of −3.75 (p = 0.0006). By 3 and 4 weeks of age, *Jag1* expression remains decreased in the mutant livers but the values are no longer statistically significant. This finding correlates temporally with the onset of fibrosis and proliferation of other cell types that are able to express *Jag1* because they are not derived from hepatoblasts.

### Jag1 and Ddr1 Interaction in Postnatal Liver

**Figure 4:**
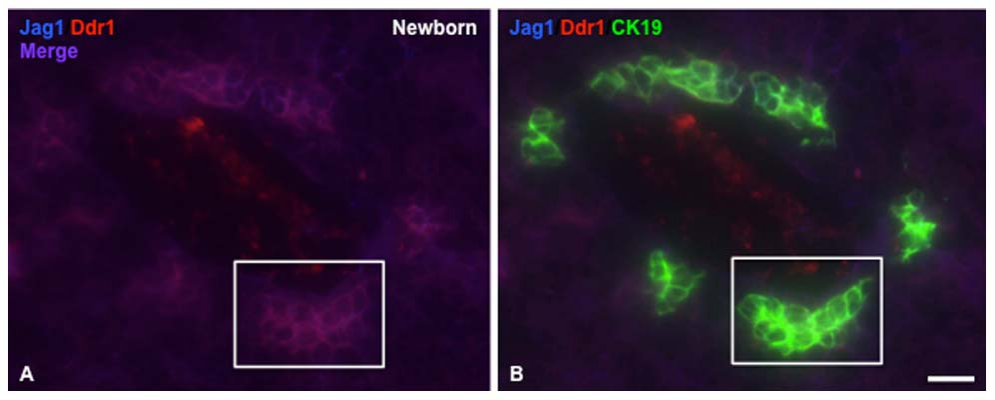
Jag1 and Ddr1 are co-expressed in newborn mouse liver. A. Immunofluorescence staining shows co-localization of Jag1 (blue) and Ddr1 (red) proteins in immature biliary cells surrounding the portal vein in C57Bl/6J mouse liver. B. Cytokeratin 19 staining (CK19, green) identifies the cells expressing Jag1 and Ddr1 as being of biliary origin. (Scale bar in [B] = 25 μ for [A] and [B]).

**Figure 5:**
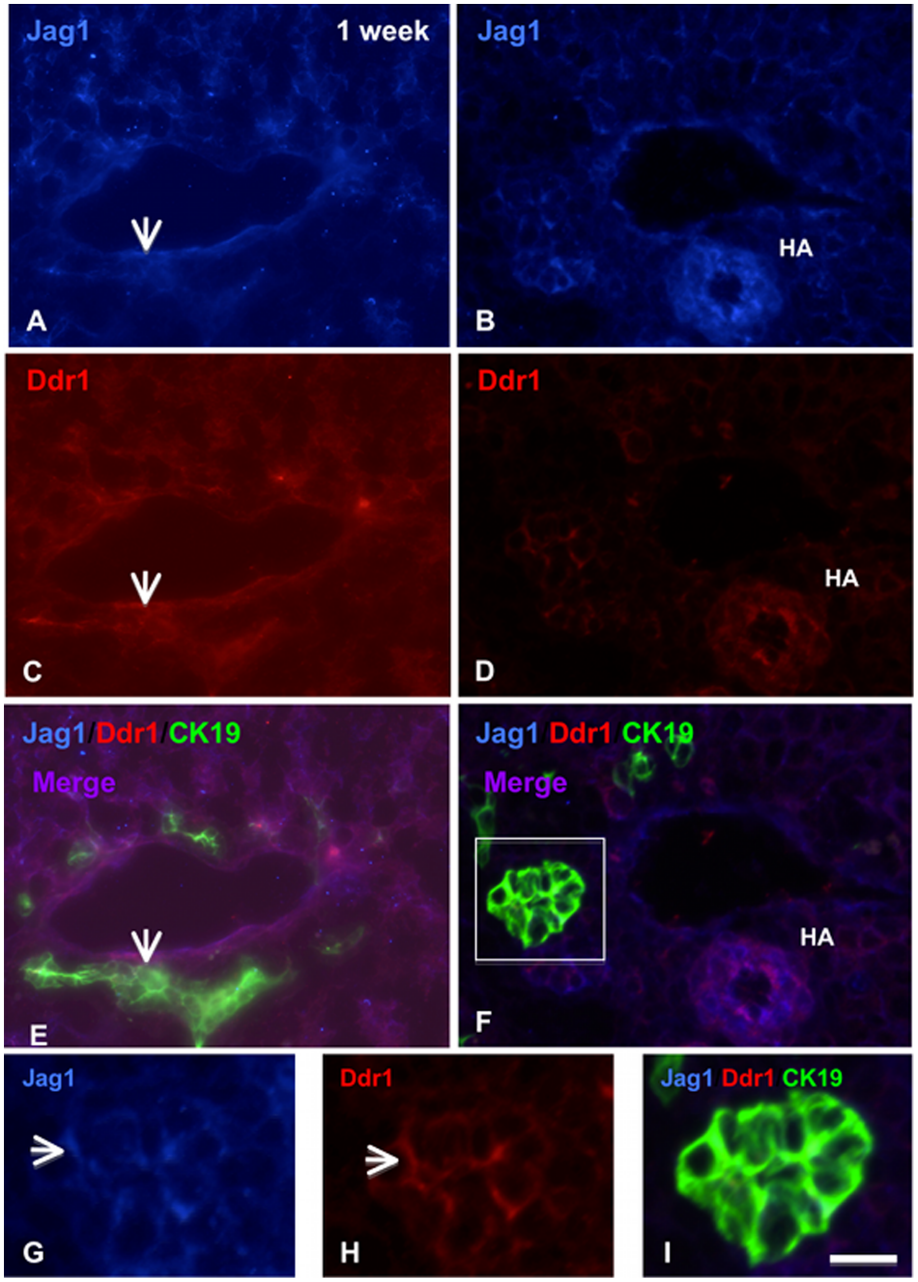
Jag1 and Ddr1 are co-expressed in mouse liver at 1 week of age. A, C, E. Jag1 and Ddr1 are co-expressed in the portal mesenchyme in C57Bl/6J mouse liver at 1 week of age, in close proximity to the bile duct (arrow). B, D, F. Jag1 and Ddr1 are also co-localized in the hepatic artery at 1 week of age in the mouse liver. G, H, I. High magnification view of the bile duct (boxed area in F) shows expression of Jag1 and Ddr1 in the intercellular junctions (arrows, G and H). (Scale bar in [I] = 25 μ for [A] through [I]).

**Figure 6:**
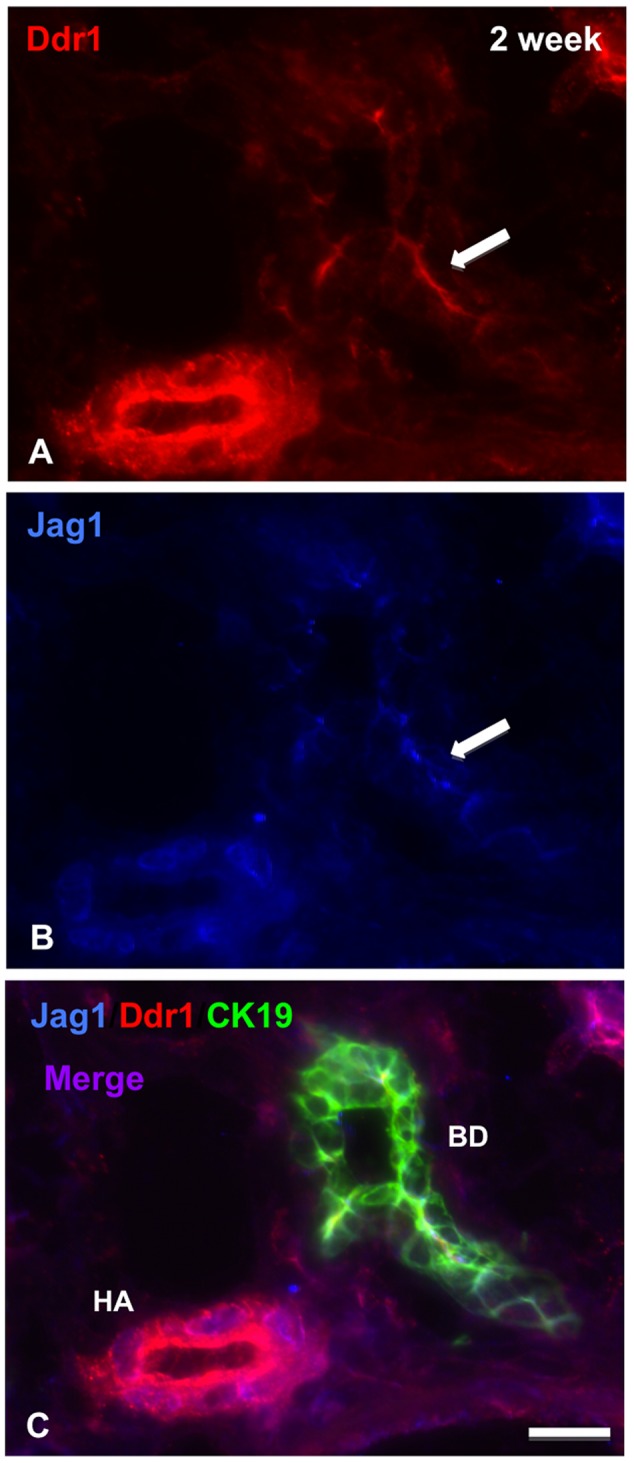
Jag1 and Ddr1 are co-localized in hepatic artery and bile duct at 2 weeks of age. A, B, C. Ddr1 (red) and Jag1 (blue) are expressed in hepatic artery in the mouse liver at 2 weeks of age (HA, panel C). While Jag1 is highly expressed in the endothelium, Ddr1 expression appears to be confined to the smooth muscle wall of the blood vessel. Ddr1 and Jag1 are both expressed in areas of intercellular junctions within bile ducts (arrows, panels A and B). (Scale bar in [C] = 25 μ for [A] through [C]).

Given that *Ddr1* was one of the most highly up-regulated genes in the *Jag1* conditional/null mutant livers, and the fact that the literature supports overlapping functions for these two genes, we investigated the expression patterns of Jag1 and Ddr1 in normal postnatal liver by immunofluorescence, and found that these two proteins are extensively co-expressed. In the newborn liver, both Jag1 and Ddr1 are expressed in biliary cells (Figure 4A, box), which are still incompletely remodeled and resemble the ductal plate in appearance. By 1 week of age, both Jag1 and Ddr1 are expressed in the portal mesenchyme (Figure 5A, C, arrows), which is in very close proximity to the bile duct (Figure 5E, arrow). Both proteins are also expressed in the hepatic artery (HA; Figure 5B, D, F) and the bile duct (Figure 5F). At a higher magnification, Jag1 and Ddr1 protein are localized to the cell junctions within the bile duct (Figure 5G, H, I). Strong expression of both proteins continues at 2 weeks in the hepatic artery and bile ducts (Figure 6A, B, C). Specifically, Jag1 and Ddr1 are co-localized at cell junctions between biliary cells (Figure 6A, B, arrow). The extensive co-expression and subcellular co-localization of Jag1 and Ddr1 suggests that these two proteins may interact directly in the postnatal liver.

**Figure 7:**
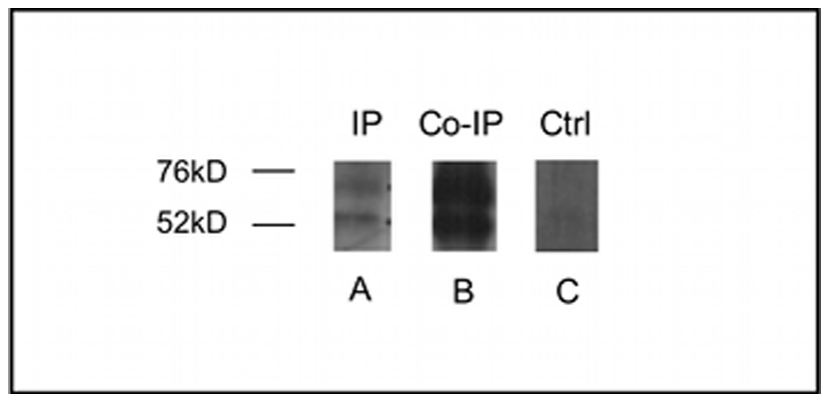
Co-immunoprecipitation of Jag1 and Ddr1. A. Immunoprecipitation of Ddr1 reveals two primary bands corresponding to the Ddr1 subunits, visualized at 54-(membrane-bound) and beta-subunits (soluble) of Ddr1. B. Coupling of Jag1 antibody to the aminolink resin and probing of western blot with Ddr1 antibody reveals co-immunoprecipitation of Jag1 and Ddr1 in mouse liver, with strong bands visualized at 54 kDa and 63 kDa. C. As a control experiment, Tie-2 antibody was coupled to the aminolink resin. Probing the western blot with Ddr1 antibody reveals no specific bands.

In order to demonstrate interaction between Jag1 and Ddr1, we performed immunoprecipitation and co-immunoprecipitation experiments using whole liver lysate from C57Bl/6J mice at 2 weeks of age. In the immunoprecipitation experiment, Ddr1-coupled resin pulled down the 63 kD alpha subunit and the 54 kD beta subunit of the Ddr1 protein (Figure 7A). The same two bands were visible when Jag1-coupled resin was used in a co-immunoprecipitation experiment, probing the membrane with Ddr1 antibody (Figure 7B). In the control lane (Figure 7C), an irrelevant Tie-2 antibody was used for the immunoprecipitation and the membrane was again probed with Ddr1 antibody, with no visible bands. In the opposite experiment, Ddr1-coupled resin was used to co-immunoprecipitate Jag1. Probing the membrane with Jag1 antibody did not demonstrate a band consistent with full length Jag1, but did reveal two smaller bands (∼40 kD and ∼52 kD), consistent with Jag1 degradation products ([17]; data not shown). To date, collagen proteins are the only known ligands for Ddr1. Further studies will be required to determine whether Jag1 can act as a functional ligand for Ddr1.

## Discussion

In this study, we have identified up-regulated expression of ECM- and fibrosis-related genes in *Jag1* conditional/null mouse livers by microarray analysis. These findings were validated by real time PCR on a custom PCR array, and expanded analysis using a targeted array showed widespread increases in expression of ECM-related genes in mutant livers at 4 weeks of age. Interestingly, these changes in gene expression resolved completely by 12 weeks of age. In addition, we have identified a novel protein interaction between Jag1 and Ddr1, which may have relevance to tissue remodeling and repair.

As we interrogated the literature for information about the differentially expressed genes identified in the microarray experiment, several lines of evidence suggested that Ddr1 would be an intriguing candidate for in depth study. First, Ddr1 encodes a receptor tyrosine kinase activated directly by collagen ligands, implying a role in cellular communication with the extracellular matrix. In fact, Ddr1 has been implicated in cell migration and ECM remodeling during branching tubulogenesis in mammary gland and bronchial development [5], both of which share similarities with biliary morphogenesis. Ddr1 has not been studied extensively in the liver, but overexpression of Ddr1 has been detected in diseased human liver [18] and in early recurrence of hepatocellular carcinoma [19]. Interestingly, Ddr1 has been implicated in a number of different human cancers, possibly enhancing cell proliferation via direct activation of canonical Notch1 signaling [11]. Also of interest is the finding that Ddr1 was identified as a potential cell surface marker for hepatic progenitors in a microarray study of rat liver [20]. Furthermore, mice lacking Ddr1 demonstrate inner ear defects similar to those seen in the Headturner mouse (Htu), a Jag1 loss of function mutant [8], [10]. Finally, Ddr1 was highly and significantly upregulated in the Jag1 conditional/null mutant livers at 4 weeks of age, the timepoint with the most dramatic phenotype of fibrosis and cellular unrest. The extensive co-localization of Jag1 and Ddr1 in postnatal liver, combined with co-immunoprecipitation data indicating protein-protein interaction and a shared domain between Jag1 and the known Ddr1 ligands, all point towards a novel protein interaction. Additionally, Ddr1 has been shown to activate canonical Notch signaling [11], suggesting that upregulation of Ddr1 might compensate for the loss of Jag1 in our conditional knockout model in the postnatal period. Given the temporal-spatial expression patterns of these two genes, it is likely that they are actively involved during biliary and arterial development and remodeling.

Precedent exists in the literature for Jag1 to interact with proteins outside the canonical Notch signaling pathway. For example, the ECM glycoprotein MAGP-2 has been found to interact with Jag1 and induce its shedding from the cell surface [21]. In addition, MAGP-2 promotes angiogenesis by antagonizing Notch signaling in endothelial cells through effects on Jag1 [22]. Thus, MAGP-2 is able to directly modulate Notch signaling via Jag1, and to a lesser extent, other Notch pathway ligands. The effect of the interaction between Jag1 and Ddr1 is as yet uncharacterized, but it is possible that Ddr1 may induce shedding of soluble Jag1 protein into the extracellular matrix. Soluble Jag1 has been found to have effects on cellular differentiation in epithelial keratinocytes [23]. Alternatively, Jag1 may act as an alternative ligand for Ddr1, with resultant effects on expression of downstream genes. Collagen, the primary ligand for Ddr1, has also been shown to interact directly with the Notch ectodomain to repress canonical Notch signaling [24]. It is certain that the Jag1/Ddr1 interactions are complex and likely to result in a myriad of downstream alterations in gene expression within the microenvironment of the extracellular matrix. Further studies will be required to fully elucidate these novel functions.

In this study, we demonstrate marked upregulation of ECM-related genes in the Jag1 conditional/null mutant livers at 4 weeks of age that is completely resolved by 12 weeks of age (Table 3). In agreement with this finding, Sirius red staining of liver tissue in these animals shows portal expansion progressing to bridging fibrosis by 4 weeks of age (Figure 2), but at later time points fibrosis is minimal [4]. These data suggest that the loss of Jag1 early in development results in a cascade of events causing activation of pro-fibrogenic pathways, but the process does not appear to be progressive. One possible explanation for the resolution of fibrosis over a series of weeks is that Jag1 expression levels are increased in other cell types, as shown by the gradual increase in Jag1 expression over the first 4 weeks in the mutant animals (Figure 3). Both Jag1 and Ddr1 have been identified as key components of wound healing and smooth muscle cell migration [7], [25], [26]. Specifically, Jag1 expression increases upon vascular injury, and a soluble form of Jagged1, presumed to act in a dominant negative fashion, had a negative effect on cell-matrix adhesion and cell migration in an *in vitro* injury model [26]. Cadherin-mediated intercellular junctions and focal adhesion complexes were also altered in the cells overexpressing soluble Jagged1. Interestingly, both Jag1 and Ddr1 are expressed in biliary cell junctions in mouse liver at 1 and 2 week timepoints (Figures 5 and 6). In addition, Ddr1 has been found to play a crucial role in smooth muscle cell migration, proliferation and neointimal thickening following arterial injury [7]. Ddr1 was also found to enhance epithelial repair in a human bronchial epithelial cell model through increased expression of matrix metalloproteinases [27]. Jag1 and Ddr1 are also both involved in regulating E-cadherin, a gene known to affect cell migration, invasion and epithelial-to-mesenchymal transition [28]–[30]. It is therefore possible that Jag1 and Ddr1 may play a role in reversal of fibrosis and downregulation of ECM-related genes at later timepoints in our mutant model.

In summary, we have presented microarray results, expression data and co-immunoprecipitation experiments that suggest a novel protein interaction between the Notch ligand Jag1 and the receptor tyrosine kinase Ddr1. The nature of this interaction and its functional consequences are as yet unclear, but further studies will be required to investigate the implications for bile duct remodeling and repair of liver injury.
