# Correction: Microarray Data Reveal Relationship between Jag1 and Ddr1 in Mouse Liver

**DOI:** 10.1371/annotation/5187e535-e7d4-4abc-b9e9-a072b04deb95

**Published:** 2014-01-24

**Authors:** Lara A. Underkoffler, Erikka Carr, Anthony Nelson, Matthew J. Ryan, Reiner Schulz, Kathleen M. Loomes

The name of the last author is spelled incorrectly. The correct name is: Reiner Schulz. 

The correct Citation is: Underkoffler LA, Carr E, Nelson A, Ryan MJ, Schulz R, et al. (2013) Microarray Data Reveal Relationship between Jag1 and Ddr1 in Mouse Liver. PLoS ONE 8(12): e84383. doi:10.1371/journal.pone.0084383.

